# Influence of food packaging color and foods type on consumer purchase intention: the mediating role of perceived fluency

**DOI:** 10.3389/fnut.2023.1344237

**Published:** 2024-01-18

**Authors:** Jie Su, Shuqi Wang

**Affiliations:** ^1^School of Economics and Management, Wuhan University, Wuhan, China; ^2^Affiliated Mental Health Center and Hangzhou Seventh People’s Hospital, Zhejiang University School of Medicine, Zhejiang University, Hangzhou, China

**Keywords:** color, food packaging, food decision making, perceptual fluency, sensory marketing

## Abstract

Packaging color entices and influences consumer perceptions and significantly affects the identification of products. Marketers manipulate the exterior packaging to influence consumer expectations, experiences, and behaviors. Building upon psychological literature on colors and emotions, we explored the influence of food packaging color and food type on consumers’ purchase intentions. Study 1 explored the interaction effects between food packaging color (warm vs. cold) and food type (vice foods vs. virtue foods) on consumers’ purchase intentions. Study 2 examined whether perceived fluency mediates the interaction effect of food packaging color and food type on consumers’ purchase intentions. The results showed that for vice foods, characterized by tastiness but unhealthiness, the utilization of warm-colored food packaging enhances consumer purchase intent. In contrast, for virtue foods that are healthful but lack gustatory appeal, the use of cold food packaging colors will lead to higher consumer purchase intent. Perceived fluency mediates the interaction effect of food packaging color and food type on consumers’ purchase intentions. This study will assist marketers to exploring a range of possibilities for packing color, impacting both the physiological and cognitive dimensions of consumer behavior related to food products, and offering practical implications for market managers.

## 1 Introduction

Human perception is significantly influenced by color, prioritizing it at 80% over shape when observing objects, gradually diminishing to equal importance with shape after 5 min (1). Color plays a pivotal role in guiding consumer perception, evident on nearly every type of food packaging (2). Beyond its role in preserving and transporting food, packaging contributes to increased sales (3) and serves aesthetic (4) and symbolic functions for consumers (5). It substantially influences product/brand recognition (6) and helps companies establish visual identities (7), fostering connections with target audiences and gaining a competitive edge (8). Considering the massive market value, packaging represents a significant portion of material costs in the food industry, constituting a substantial portion of the GDP (9).

Due to the crucial role of color in consumer visual perception, businesses meticulously choose colors in product promotion. Inappropriate choices can misrepresent products, dissuading consumers from making purchases (10). Packaging colors play a crucial role in establishing connections between businesses and target audiences (11). They significantly alter consumers’ perceptions of food desirability and enjoyment (12). For instance, red or orange packaging is commonly used for delicious chicken wings, and altering this color to green might create discomfort due to perceptual inconsistency (13). While some businesses deliberately use inconsistent colors (e.g., green packaging for Coca-Cola) to attract consumers, this study does not focus on this pursuit of uniqueness. It aims to explore the impact of color consistency with food types on consumer purchase intentions and the underlying psychological mechanisms.

Prior research has acknowledged the influence of product packaging on consumer expectations (14) and experiences (15). However, limited research exists on the effect of packaging color (cool vs. warm tones) and various food types on consumer purchasing decisions. Food categorization into vice (delectable but unhealthy, e.g., chocolate cake) and virtue (less tasty but healthier, e.g., vegetable salads) has been adopted in food research, unraveling psychological responses such as self-control (16) and guilt (17). This study delves into the influence of consistency between food packaging color and food type on consumer purchasing decisions.

This paper has a two-fold contribution: firstly, it examines the significant impact of matching food packaging color to the food product on consumer choices. Warm-colored packaging suits vice foods, while cool-colored packaging aligns better with virtue foods. Secondly, it delves into perceptual fluency, where visually easy-to-process elements are preferred, generating positive emotions. Specifically, warm-colored packaging for vice foods and cool-colored packaging for virtue foods enhance purchasing behavior through perceived fluency.

The study aims to investigate the effects of color (warm vs. cold) and food type (vice vs. virtue) on consumer purchase intentions. The subsequent sections present the theory and conceptual framework, with two studies validating the underlying conceptual framework. Study 1 explores the interaction effect of food type and color type on consumers’ purchase intentions. Study 2 validates perceived fluency as a mediating process. Lastly, the paper discusses the theoretical and practical implications, offering insights for future research directions.

## 2 Theoretical background and hypotheses development

### 2.1 Role of food packaging and color

People are constantly surrounded by an array of colors in their daily lives (18), establishing color as a fundamental aspect of human perception (19). Color serves as both a conduit for conveying information (20) and an aesthetic tool (21). It can be defined across various dimensions (e.g., hue, saturation, lightness) (22). In the 1940s, Swiss psychologist Max Luscher classified colors along the spectrum of cool and warm tones (21), categorizing hues into two groups: cool and warm. The field of food packaging has extensively studied color (16). Relevant studies have delved into how packaging color influences consumer perceptions (23), emotions (24), and purchase intentions (25). Some research explores the relationship between packaging color and product attributes (26). For instance, warm-colored packaging might closely associate with the tastiness or sweetness of food, while cool colors might relate to the freshness or health attributes of the product (27). The significant influence of color on taste perception suggests that consumers can perceive taste changes solely based on visual cues (27). Indeed, changes in the color of utensils used (28), serving plates (29), and product packaging (30) can markedly affect the perceived taste of food items (31). For instance, Koch and Koch (32) discovered a negative correlation between red color and sour, bitter, and salty tastes. Chylinski et al. (33) studied variations in the perceived intensity of sweetness associated with changes in color saturation, particularly focusing on the color red. This study aims to investigate the correlation between food packaging color and food type and its impact on consumer purchase intention, specifically considering food packaging color (cool and warm).

### 2.2 Food types and taste associations

“Vice” and “Virtue” are frequently used in discussions regarding dietary habits, nutritional choices, and health-conscious eating patterns (34). These terms aid in categorizing foods based on their impact on health and nutrition, assisting individuals in making informed dietary choices (35). Previous literature has categorized foods into “virtue food” and “vice food” based on the balance between their costs and benefits (36). Vice foods are often considered indulgent, delicious, or highly palatable but might lack nutritional value or contain higher levels of sugars, fats, or processed ingredients (desserts, fried foods, sugary treats, fast food) (37). On the other hand, virtue foods are generally perceived as healthier, more nutritious, and beneficial for overall well-being (34). These foods are often lower in calories, have high nutritional value, and are associated with promoting health (fruits, vegetables, whole grains, lean proteins).

From an evolutionary psychology perspective, it has been observed that humans have gradually developed a preference for warm-colored foods throughout evolution. Immature fruits often exhibit sourness and display cool colors like green, while as they ripen, they transition to warm colors such as red and orange (38), indicating higher sugar or fat content (39). Consequently, warm-colored foods are associated with increased sweetness and higher fat content. Foods high in sugar and fat tend to evoke pleasure and elicit positive emotional responses after consumption (40). Consumers tend to derive meaning from the color of products (40). Similarly, perceptions of different packaging colors vary among consumers (41). For instance, studies suggest that desserts packaged in black (compared to white or yellow) are expected to contain more chocolate (30). Based on these insights, we hypothesize the following:

*H1*: Vice foods packaged in warm colors lead to higher purchase intentions of consumers relative to cold colors.

According to Raghunathan et al. (42), people believe that a food’s taste and healthiness are inversely related. Consequently, consumers tend to perceive unhealthy products as inherently tastier than their healthier counterparts. Huang and Lu (43) consider products in blue packaging as healthier and more likely to be purchased than products in red packaging. Meanwhile, Clarke and Costall (44) analyzed the associations between colors and emotions. Their results showed that cool colors (e.g., blue and green) evoke sedative emotions. Relying on a judgment heuristic (45), there is an association between cold-colored (green, white) food packaging and organic food. Consequently, encountering seemingly virtue food in cold-colored packaging activates this heuristic, eliciting positive health associations due to its association with a reduction in certain food components (e.g., low-fat or low-sugar). Thus, we hypothesized the following:

*H2*: Virtue foods packaged in cold colors lead to higher purchase intentions of consumers relative to warm colors.

### 2.3 Perceived fluency

Marketers have a tendency to infuse unconventional visual cues into packaging design (46), leveraging unique visual elements to capture consumer attention (47). When making judgments, individuals do not solely rely on thoughts but also on the metacognitive experience of processing these thoughts (48). An array of studies has demonstrated the impact of contextual cues and environmental guidance on consumer judgment (49).

Perceptual fluency refers to the cognitive experience of how easily or rapidly individuals process an object or information (49). It encompasses identifying, comprehending, and processing information, involving the acquisition and processing of data from sensory channels like visual and auditory inputs (50). Low-level perceptual fluency links with physical attributes (such as ease in identifying colors, shapes, or patterns), while high-level fluency involves semantic meanings (like understanding words, language, or symbols) (51). Higher perceptual fluency implies easier, faster information processing, generally resulting in more positive emotions and preferences (52). When a person effortlessly recognizes or identifies a stimulus without errors, it indicates high perceptual fluency (53). Thus, perceptual fluency heightens when stimulus features (like color or value contrast) assist in consumer processing (54).

Perceptual fluency is extensively discussed in psychology and consumer behavior research. Previous studies delving into cognitive processing, consumer preferences, packaging colors, and emotions contribute to a deeper understanding of how perceptual fluency influences consumer decisions and emotional experiences. Huang and Lu (43) suggested warm colors might be closely linked to a product’s sweetness, deliciousness, or emotional appeal, while Barrett et al. (55) associated cool colors with a product’s freshness or health attributes. Consumers often prioritize easily processed cue information when forming judgments and decisions (56). Reber et al. (58) discovered that variables facilitating perceptual processing enhance consumer preference for a stimulus. Vision significantly influences other senses, notably taste (57, 58). As per the resource matching theory, information processing efficiency peaks when utilized resources align optimally with task requirements (59).

We propose that for tempting vice foods, warm-colored packaging enhances perceptual fluency more than cold-colored packaging. Conversely, for wholesome virtue foods, cold-colored packaging enhances perceptual fluency more than warm-colored packaging. Consequently, we anticipate perceived fluency to mediate the impact of package color and food type on consumer purchase intentions. Hence, we hypothesize the following:

*H3*: Perceived fluency mediates the effect of food package color and food type on consumer purchase intention.

**Figure 1:**
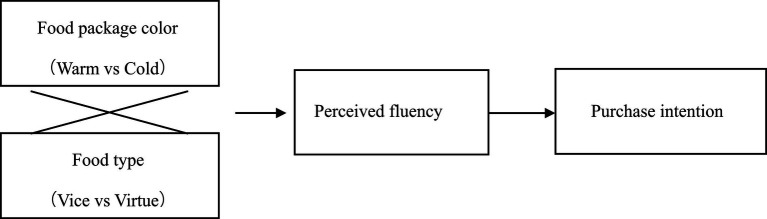
Theoretical model.

The research model is presented in Figure 1. We empirically examine our hypotheses through two studies. Study 1 was an experimental study that explored the impact of food package color and food type on consumer purchase intention (H1, H2). Study 2 aims to tests the mediating role of perceived fluency (H3).

## 3 Study 1 package color and food type effects on consumers’ purchase intention

Study 1 aimed to provide an initial demonstration of our predictions concerning food package colors and food types. In this study, we examined consumers’ purchase intentions by manipulating food packaging colors and food types. While the conventional use of dessert as vice and salad as virtue aligns with previous research practices (60), a pre-test was conducted on the experimental materials used in Study 1 before the main study to ensure the robustness of the findings.

### 3.1 Pre-test

Before initiating the formal study, we conducted a pre-test to validate the efficacy of the stimuli used in our study. Fifty participants from the online platform (M_age_ = 37.52, SD = 14.749, 46% female) responded to a question adapted from Khan and Dhar (61). Specifically, participants were informed, “A virtue is less tasty but good for future health. A vice food is tasty but not good for health.” Participants then rated either a salad or a dessert on a scale of 0 to 100 (0 = vice; 100 = virtue).

As expected, a paired samples t-test confirmed that participants rated the vice (vs. virtue) group of food as more vice (vs. virtue) on the 100-point scale [M_vice_ = 32.88, SD = 18.807; M_virtue_ = 71.08, SD = 16.393; *t* (50) =7.565, *p* < 0.000]. Thus, there was a notable difference in vice–virtue perceptions among participants.

### 3.2 Method

Two hundred and twelve undergraduate students from a university in western China participated in the study for course credit. After excluding participants who did not complete the questionnaire, a total of two hundred and four participants were retained (M_age_ = 20.75, SD = 4.359, 44.6% female). The participants were randomly divided into four groups, determined by a 2 (package color: warm vs. cold) × 2 (food type: vice vs. virtue) between-subjects design. Following the color designations used by Huang (2022), we defined orange as the warm color and green as the cool color.

**Figure 2:**
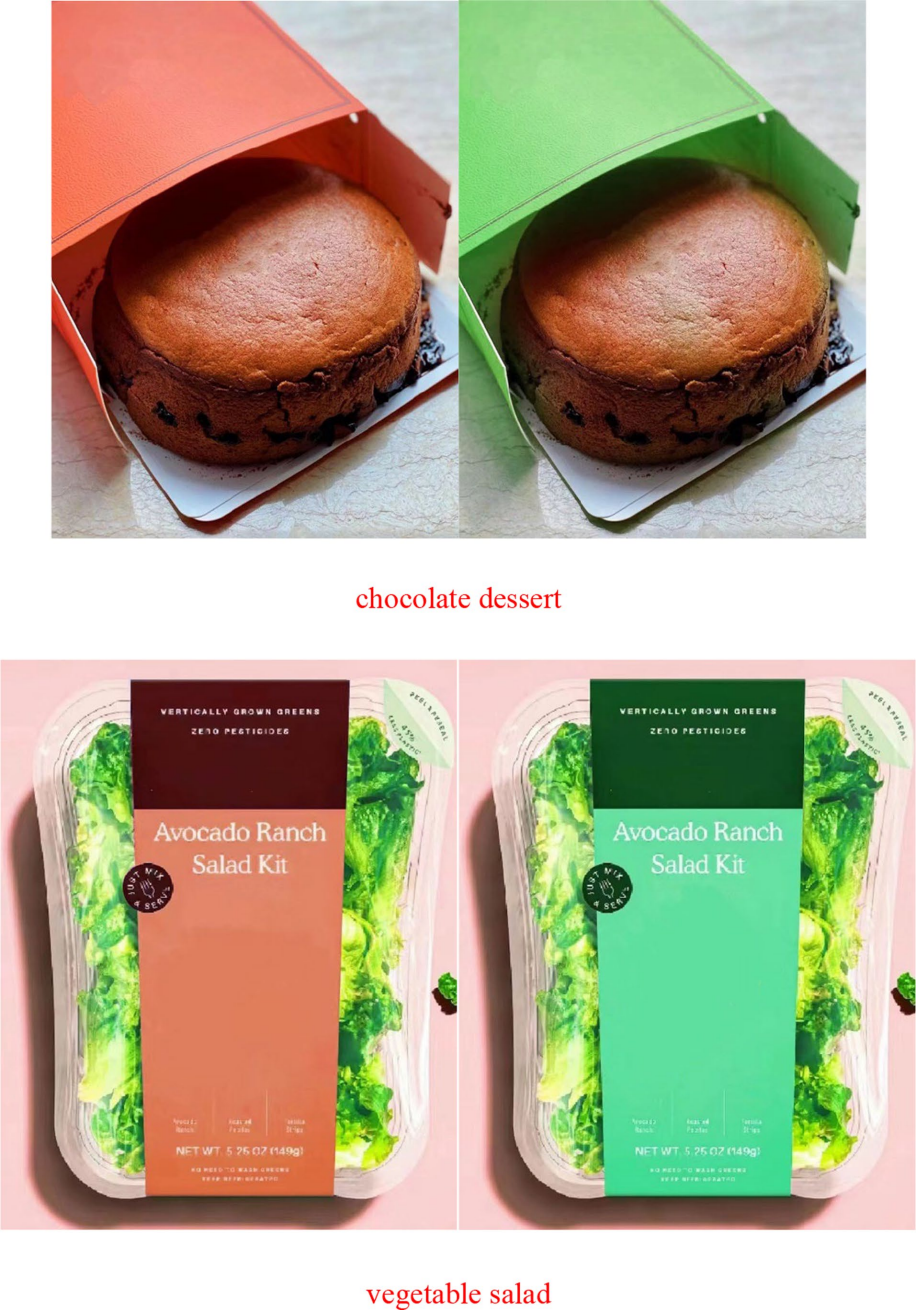
Chocolate dessert and vegetable salad.

The participants were asked to read “Imagine that you are shopping in the supermarket to buy a chocolate dessert/vegetable salad, and you come across the cake/frozen section where you see a chocolate dessert/vegetable salad in a package.” Following these instructions, participants were shown a picture of a chocolate dessert/vegetable salad packaged in different colors (Figure 2). Subsequently, on the next screen, further instructions were provided, “We would like to know, how likely are you to buy chocolate dessert/vegetable salad packaged in this color.” (1 = very unlikely to buy, 7 = very likely to buy); adapted from Landwehr et al. (62).

Afterwards, participants were presented with the definitions of vice food and virtue food (aligned with the pre-test) and were then prompted to rate either a salad or a dessert on a 0 to 100 scale. Finally, participants answered demographic questions.

### 3.3 Result

#### 3.3.1 Manipulation check

A paired samples t-test confirmed that participants rated the vice (vs. virtue) group of food as more vice (vs. virtue) on the 100-point scale [M_vice_ = 42.57, SD = 21.451; M_virtue_ = 72.44, SD = 23.092; *t* (204) =9.572, *p* < 0.000]. Consequently, there was a significant difference in vice–virtue perceptions, aligning with our expectations, indicating the successful manipulation of the type of food.

#### 3.3.2 Purchase intention

**Figure 3:**
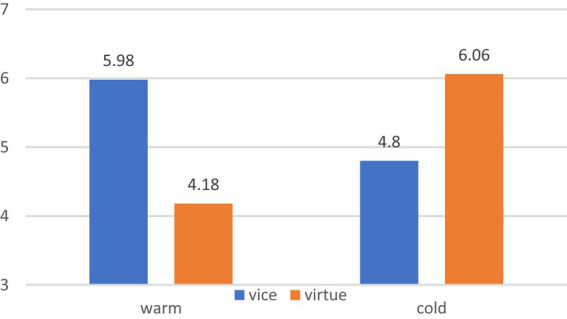
The interaction effect of food type and package color.

A 2 (food type) × 2 (package color) ANOVA showed no significant main effect of food type [*F* (1,204) = 2.037, *p* = 0.155] and package color [*F* (1,204) = 3.368, *p* = 0.068]. However, a significant interaction was observed between food type and package color [*F* (1,204) = 63.243, *p* = 0.000]. Specifically, for vice foods, warm (vs. cold) package colors increased the purchase intention [M_warm_ = 5.98, SD = 1.029; M_cold_ = 4.80, SD = 1.600; *F* (1,204) = 19.497, *p* = 0.000]; for virtue foods, cold (vs. warm) package colors increased the purchase intention [M_warm_ = 4.18, SD = 1.819; M_cold_ = 6.06, SD = 0.785, *F* (1,204) = 46.063, *p* = 0.000]. These findings support and validate H1 and H2, as depicted in Figure 3.

## 4 Discussion

Study 1 provides initial evidence supporting the interaction effect of food type and package color on consumer purchase intention. Specifically, warm packaging is more likely to enhance consumer purchase intentions for vice foods, whereas cold packaging is more likely to increase consumer purchase intentions for virtue foods, validating Hypotheses 1 and 2. To further explore the underlying mechanism behind this effect, Study 2 will be conducted to investigate the mediating factors involved.

## 5 Study 2 the mediating role of perceptual fluency

Study 2 was designed with three primary objectives. Firstly, the study aimed to establish causal evidence supporting the impact of food type and package color on consumers’ purchase intention. Secondly, to eliminate potential influences from food preferences and brand differences, which were present in Study 1 where desserts and salads were used, Study 2 employed the same food category—cereals (whole grain cereals and chocolate cereals) (63) to further test our hypothesis. Thirdly, we examined whether perceptual fluency mediates the effect of food type and package color on consumers’ purchase intention. In order to ensure the robustness of the study, a pre-test was conducted to examine the experimental materials used.

### 5.1 Pre-test

In a pre-test, we recruited 50 participants from the internet (M_age_ = 32.44, SD = 6.987, 44% female). Initially, participants read the definitions of “vice” and “virtue” foods and then responded to a question identical to that in Study 1. The results showed that whole grain cereals were perceived as more virtuous, whereas chocolate cereals were perceived as more vice [M_vice_ = 27.88, SD = 14.630; M_virtue_ = 66.64, SD = 15.058; *t* (50) =13.734, *p* < 0.000]. Thus, there was a notable difference in vice–virtue perceptions among participants.

### 5.2 Method

A total of 252 participants were initially recruited from the internet, with four excluded due to incomplete questionnaire responses, resulting in an enrollment of 248 participants (M_age_ = 30.65, SD = 8.162, 50.2% female). Participants were randomly assigned to a 2 (package color: warm vs. cold) × 2 (food type: vice vs. virtue) between-subjects design. Consistent with Study 1, orange was used as the warm color, and green was used as the cool color.

**Figure 4:**
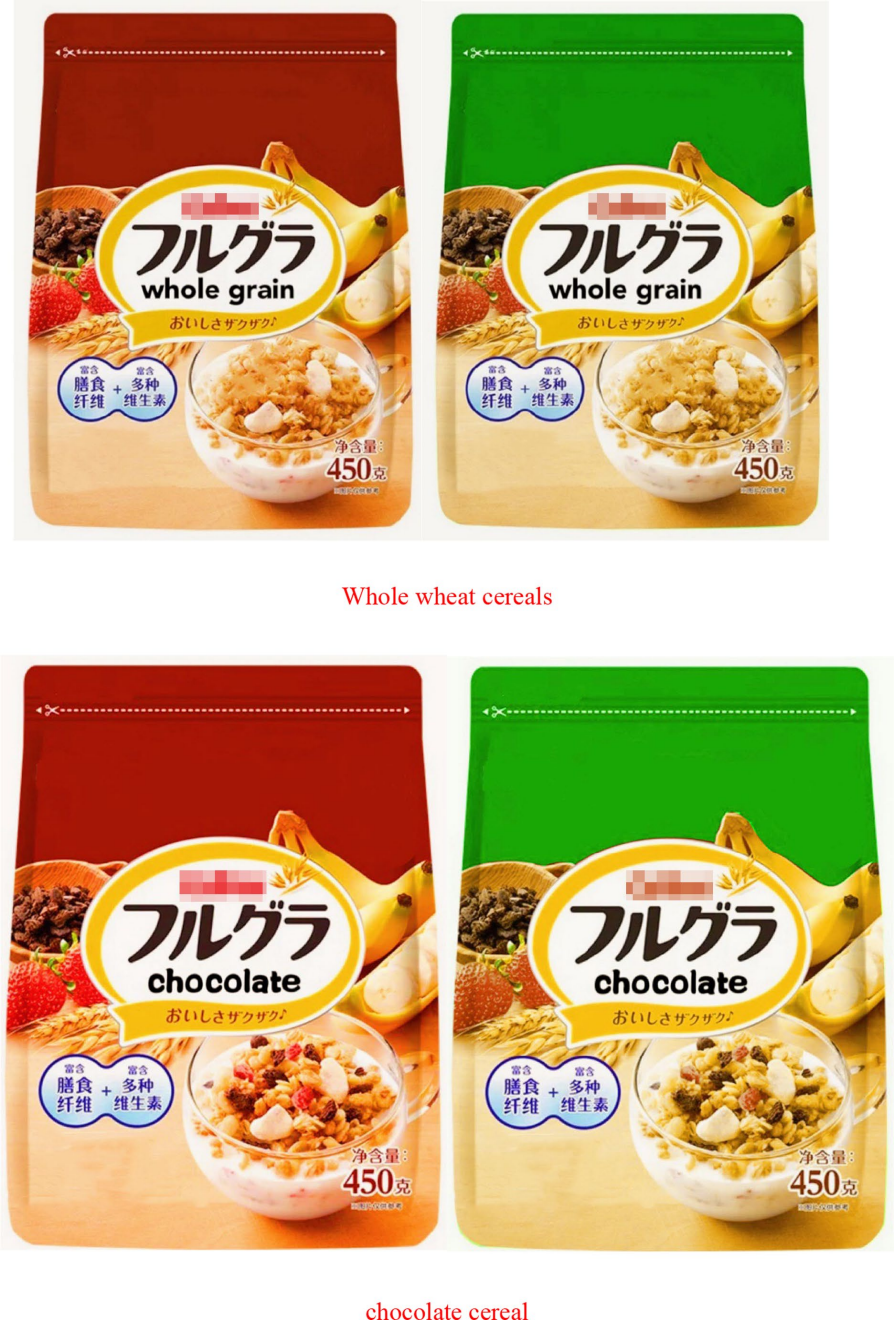
Chocolate cereals and whole wheat cereals.

The participants were asked to read “Imagine that you are shopping in the supermarket to buy a whole grain cereals/chocolate cereal and you come to the cereals section and see a whole wheat cereals/chocolate cereal in a package.” Subsequently, they viewed images of whole grain cereals and chocolate cereals packaged in various colors (Figure 4). After participants watched, the next screen provided further instructions, “We would like to know, how likely are you to buy whole grain cereals and chocolate cereals packaged in this color.”

Following this, participants responded to four items assessing perceived fluency, rating statements such as: “The color of the food packaging in the picture immediately catches your attention,” “The color of the food packaging in the picture stands out from the rest,” “The color of the food packaging in the picture is easy to associate with the type of product,” and “It is easy to identify the type of food product from the food packaging color in the picture” (*α* = 0.752). All items were rated on a 7-point scale.”

Afterward, participants read the definitions of vice food and virtue food (consistent with the pre-test) and were asked to rate both whole grain cereal and chocolate cereal on a scale from 0 to 100. Finally, the participants answered demographic questions.

### 5.3 Result

#### 5.3.1 Manipulation check

A paired samples *t*-test confirmed that participants rated the vice (vs. virtue) group of food as more vice (vs. virtue) on the 100-point scale [M_vice_ = 37.39, SD = 17.668; M_virtue_ = 72.01, SD = 23.047; *t* (248) = 32.166, *p* < 0.000]. Thus, vice–virtue perceptions differed as expected, indicating the successful manipulation of the type of food.

#### 5.3.2 Purchase intention

**Figure 5:**
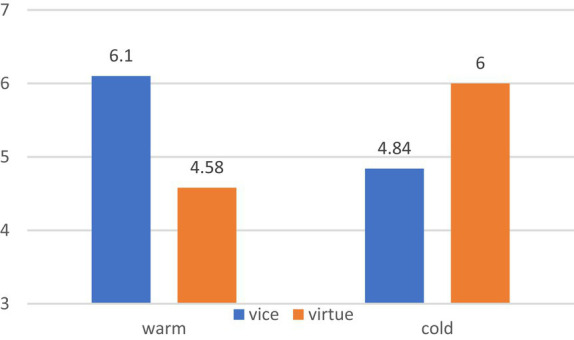
The interaction effect of food type and package color.

A 2 (food type) × 2 (package color) ANOVA showed no significant main effect of food type [*F* (1,248) = 0.305, *p* = 0.581] and package color [*F* (1,248) = 1.475, *p* = 0.226]. However, a significant interaction was observed between food type and package color [*F* (1,248) = 83.962, *p* = 0.000]. Specifically, for vice foods, warm (vs. cold) package colors increased the purchase intention [M_warm_ = 6.10, SD = 0.863; M_cold_ = 4.84, SD = 1.308; *F* (1,248) = 39.957, *p* = 0.000]; for virtue foods, cold (vs. warm) package colors increased the purchase intention [M_warm_ = 4.58, SD = 1.477; M_cold_ = 6.00, SD = 0.810, *F* (1,248) = 44.016, *p* = 0.000]. These results validate H1 and H2, as depicted in Figure 5.

#### 5.3.3 Mediation effects

To examine the mediating role of perceptual fluency, the mediating variables were examined using the bootstrap method, referring to the moderated mediation model proposed by Hayes (64). We tested this indirect effect using Model 8 in PROCESS, with 5,000 bootstrapped samples. The results indicated a significant indirect effect of perceptual fluency [*β* = 0.0944; SE = 0.0521; CI = (0.0129, 0.2131)]. Specifically, for warm-colored packages of vice foods, the mediating effect was significant [*β* = 0.0720; SE = 0.0373; CI = (0.0140, 0.1559)]; for cold-colored packages of virtue foods, the mediating effect was significant [*β* = 0.223; SE = 0.0301; CI = (0.0915, 0.0302)]. Thus, perceptual fluency mediated the interaction effect of vice food and the warm color package on the purchase intention, confirming H3.

### 5.4 Discussion

The results from Study 2 confirm that warm-colored packaging increases purchase intentions when the food is vice, while cool-colored packaging enhances purchase intentions when the food is virtue, which aligning with hypotheses H1 and H2. We further investigated the mediating role of perceived fluency. It was found that for warm-colored packaging with vice foods and cool-colored packaging with virtue foods, perceived fluency mediated the impact of food type on purchase intentions for different package colors, supporting Hypothesis H3. Finally, in Study 2, various independent variables were employed to enhance external validity.

## 6 General discussion

Food providers employ a diverse range of elements in their marketing arsenal, encompassing various package components. This study focuses on a pivotal and commonly utilized visual design element in this context: package color. These elements span explicit features like color and shape to more nuanced design elements. Drawing from psychological insights, our research delved into how food package color, combined with the type of food, influences consumers’ intentions to purchase. Across two studies involving distinct participant groups (college students and adults online) and utilizing manipulated food types through real food images, we discovered that vice foods presented in warm-colored packaging led to increased purchase intentions. Conversely, virtue foods exhibited in cool-colored packaging resulted in heightened purchase intentions (Study 1). Our investigation revealed that this effect was mediated by perceptual fluency (Study 2).

### 6.1 Theoretical implications

This research makes several contributions to the marketing literature. Firstly, our research adds to the existing body of packaging literature in marketing. Prior research has explored various dimensions of product packaging design, emphasizing its crucial role in shaping consumer judgments and decisions. For instance, research has delved into aspects of graphic design in product packaging, examining the impact of pale versus bright coloring or high versus low image placement on consumers’ purchase intentions and willingness to pay (65–67, 70). Our contribution lies in investigating how packaging color—warm versus cold—alters consumers’ assessments of packaging and how these evaluations influence product purchase decisions.

Secondly, our research contributes to the fluency literature. Earlier studies have demonstrated that semantic primes can facilitate the processing of conceptually related visual stimuli. For instance, Winkielman and Fazendeiro (67) observed that exposure to the word “key” facilitated the processing of a picture of a lock, resulting in more favorable evaluations of the lock. Similarly, consumers evaluate food based on its taste, healthiness, freshness, and even the packaging, among other factors. In food purchases, consumers encounter the food packaging first, suggesting that the color of food packaging influences the consumer’s assessment of the food. As fluent processing is generally perceived positively (67, 68), stimuli processed fluently are considered more attractive and pleasing (69), thereby enhancing liking for a product that exhibits relevant visual features. Thus, our experiments demonstrate that the prime and the target must be meaningfully related when the prime matches the perceptual features of the target. Our results show that for vice (vs. virtue) foods, packaging in warm (vs. cool) colors can enhance perceptual fluency and consequently, consumer purchase intent.

### 6.2 Managerial implications

Color serves as a crucial informational cue for consumers, allowing marketers to influence consumer perceptions through strategic color choices. Hence, from a managerial perspective, marketers should exercise prudence when leveraging color knowledge for packaging, advertisements, and websites. Within the food industry, caution is advised for food suppliers promoting products with varied color packaging, as this can lead to divergent consumer perceptions across different food retail settings. Our research indicates that packaging vice foods in warm colors and virtue foods in cool colors leads to increased consumer purchase intentions. Therefore, it’s crucial for food providers to establish a clear positioning for their food products before devising marketing strategies. If a food is positioned as a vice food, warm-colored packaging is recommended; conversely, if the food is positioned as a virtue product, cool-colored packaging is preferable. Marketers should recognize that in-store purchase situations heavily rely on sensory properties, especially for product innovations or unfamiliar food items. It is important to highlight that the product or retail environment characteristics shape the interpretation of packaging cues. This underscores the significance of avoiding negative consumer evaluations resulting from inconsistencies with other diagnostic cues.

Regarding public policy implications, while our findings indicate that aligning packaging color with food type heightens consumer purchase intentions, particularly for indulgent foods using warm colors, excessive consumption of high-sugar, high-calorie indulgent foods pose health risks. Hence, in regions with high obesity rates, appropriately adjusting packaging colors of certain high-calorie, high-sugar vice foods could positively impact public health and well-being.

### 6.3 Limitations and further research

This work has certain limitations call for further research. Firstly, our categorization of “vice” and “virtue” foods is based on the product category level. While our research primarily contrasts vice foods versus mainly virtue foods, we have not examined mixed vice-virtue bundles. For example, whether red wine is perceived as a virtue or vice food might depend on whether its health benefits or alcoholic properties are emphasized. Future research could explore the consumer perceptions of mixed vice-virtue bundles, such as red wine packaged in cool colors.

Secondly, although our study focuses on visual cues in sensory marketing, future research could explore whether food type, when compared to other attributes, significantly influences consumer behavior. For instance, do spicy (gustatory) foods pair better with warm-colored packaging, or are soft foods (tactile) align more with warm-colored packaging?

Lastly, in practice applications, some western marketing practitioners often use inconsistent package colors to attract consumer attention. For instance, they might use green packaging for fried chicken and red packaging for salads, which contradicts our findings. Future research could further explore the influence of personality and culture in these contexts. For example, westerners, with their more independent-self personalities, might be more receptive to novel and inconsistent products. In contrast, easterners, with their interdependent-self personalities, may lean towards conservatism and be more susceptible to social influences.

## Data availability statement

The raw data supporting the conclusions of this article will be made available by the authors, without undue reservation.

## Author contributions

JS: Writing – original draft, Conceptualization, Data curation, Formal analysis, Investigation, Visualization. SW: Writing – review & editing, Conceptualization, Formal analysis, Methodology, Project administration, Validation, Visualization.
